# The data of GDP and exchange rate used in the Balassa–Samuelson hypothesis

**DOI:** 10.1016/j.dib.2016.09.044

**Published:** 2016-10-03

**Authors:** Weiguo Wang, Jing Xue, Chonghua Du

**Affiliations:** School of Economics, Dongbei University of Finance and Economics, China

**Keywords:** Real GDP per capita, Real exchange rates, Balassa–Samuelson Hypothesis, Developed countries, Developing countries

## Abstract

This article introduces the data of the log real GDP per capita ratio and the log real exchange rate which are used to revisit the Balassa–Samuelson Hypothesis. We acquired the data from IMF and World Bank database, and provide the name and source of the data. All data are openly accessible. Besides, we describe the value of data as well as the method to process the data which can also be found in “The Balassa–Samuelson Hypothesis in the developed and developing countries revisited” (Weiguo Wang, Jing Xue, Chonghua Du, 2016) [1].

**Specifications Table**

**Table:** 

Subject area	*Economics*
More specific subject area	*Macroeconomics, Econometrics*
Type of data	*Excel file*
How data was acquired	*Collected from database of IMF and World Bank*
Data format	*Analyzed*
Experimental factors	*Data were estimated using GDP per capita, CPI and nominal exchange rate obtained from the World bank and IMF database (see Data accessibility) applied to 20 developed countries in 35 years and 20 developing countries in 30 years.*
Experimental features	*Data includes log of the real GDP per capita ratio, and log of real exchange rate for each country.*
Data source location	*Developed countries: Australia, Belgium, Canada, Denmark, Finland, France, Germany, Greece, Ireland, Israel, Italy, Japan, Korea, Netherland, Norway, Portugal, Singapore, Spain, Sweden, and United Kingdom*
	*Developing countries: Algeria, Brunei Darussalam, Cameroon, Chile, China, P.R., Colombia, Dominica, Gabon, Grenada, India, Indonesia, Malaysia, Mexico, Morocco, Philippines, South Africa, Sri Lanka, St. Kitts and Nevis, St. Vincent and the Grenadines, and Venezuela, R.B.*
Data accessibility	*Data is with this article and available at*http://data.imf.org/regular.aspx?key=60998125
	http://data.imf.org/regular.aspx?key=60998120
	http://databank.worldbank.org/data/reports.aspx?source=2&series=NY.GDP.PCAP.PP.CD&country=

**Value of the data**

• This data allows researchers who are not familiar with the pretreatment of the euro-zone countries’ nominal exchange rate to gain understandings and skills of converting the euro exchange rate into the national own exchange rate.
• This data separates the developing countries from the developed countries so that the Balassa–Samuelson Hypothesis can be tested more specifically.
• This data can be accessed without restrictions that are usually placed on developing countries, which may inspire more research ideas and opportunities.

## 1 Data

CPI, nominal exchange rates and GDP per capita are raw data which are pretreated for the analysis of the Balassa–Samuelson Hypothesis. CPI and nominal exchange rates come from the International Financial Statistics (IFS) database maintained by the IMF, and GDP per capita data come from the World Bank national accounts data, and OECD National Accounts data files.

## 2 Experimental design, materials and methods

We apply real GDP per capita data in “The Balassa–Samuelson Hypothesis in the developed and developing countries revisited” [1], so we use CPI as a deflator to deflate the nominal GDP per capita acquired from the resource described above into the real GDP per capita. The year 2010 is selected as a base-year. We took log of the real GDP per capita and we used the log real GDP per capita ratio (which is the difference between the log real GDP per capita of the U.S. and the home country) as the proxy of the relative productivity, just as many researchers do in their studies [2], [3], [4]. In 1999 the Euro became a single currency in the Euro-zone. We convert the post-1999 nominal exchange rates for the euro-zone countries into national currency according to the official euro conversion rates. The log real exchange rate (RER) is calculated by sum of the log nominal exchange rate, and the difference of the log CPI of the U.S and the home countries.

As the dependent variable-RER in developed countries is non-stationary for only one case and stationary for the other three when we apply unit roots test. We use the co-integration tests to test the co-integration between two components of this dependent variable. Readers can refer to the article [1] for more details. The nominal exchange rates in developed countries, and its corresponding difference between log CPI of the U.S. and the home country, can also be found in the excel file (see online Supplementary material).
