# Supplementary material for: The data of GDP and exchange rate used in the Balassa–Samuelson hypothesis
Source: Data Brief. 2016 Oct 3;9:594–6. doi: 10.1016/j.dib.2016.09.044 (PMC5064988; doi:10.1016/j.dib.2016.09.044)
Supplement: Supplementary file 1 — Supplementary material [file mmc1.docx]

Conflict of interest: none.
